# Supplementary material for: The phase diagram and hardness of carbon nitrides
Source: Sci Rep. 2015 May 6;5:9870. doi: 10.1038/srep09870 (PMC4421826; doi:10.1038/srep09870)
Supplement: Supplementary Information — for the phase diagram and hardness of carbon nitrides [file srep09870-s1.pdf]

Supplementary Information for

The phase diagram and hardness of carbon nitrides

Huafeng Dong[1,2], Artem R. Oganov[1,2,3,4*], Qiang Zhu[1,2], and Guang-Rui Qian[1,2]

[1]*Department of Geosciences, Stony Brook University, Stony Brook, New York 11794-2100, USA*

[2]*Center for Materials by Design, Institute for Advanced Computational Science, Stony Brook University, Stony Brook, New York 11794-2100, USA*

[3]*Moscow Institute of Physics and Technology, 9 Institutskiy Lane, Dolgoprudny City, Moscow Region 141700, Russia*

[4]*School of Materials Science, Northwestern Polytechnical University, Xi'an 710072, China*

*Corresponding author. E-mail: artem.oganov@stonybrook.edu (A.R.O.)

This file includes:

Supplementary Figure 1-6

Supplementary Table 1-5

Supplementary data 1-3 | Brief descriptions of the crystal structures

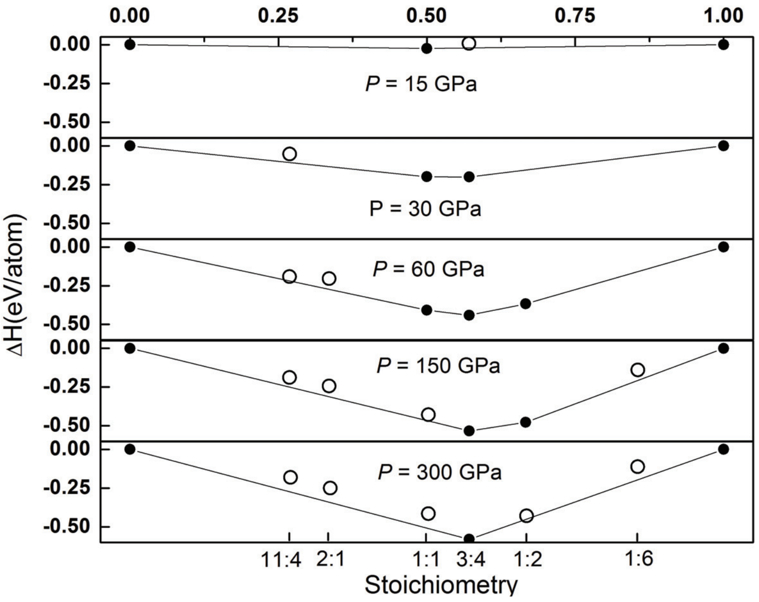

Supplementary Figure S1 | Convex hull diagram for C-N system at selected pressures.

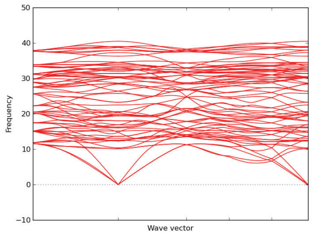

A. *I*2*d*-CN_2

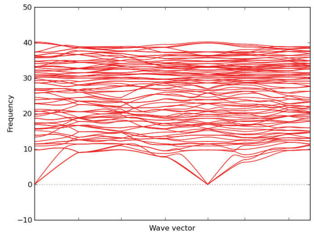

B. *P*31*c*-C_3N_4

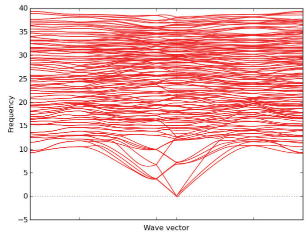

C. *Cm*-C_3N_4

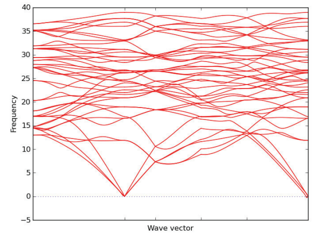

D. *I*2*m*-C_3N_4

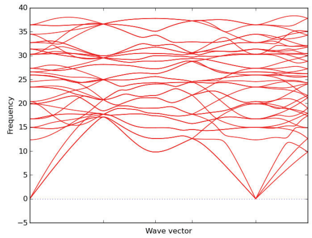

E. *I*3*d*-C_3N_4

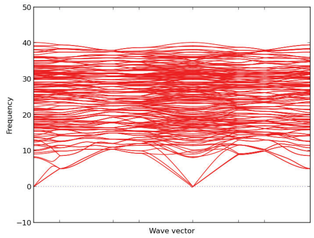

F. *Cmc*2_1-C_3N_4

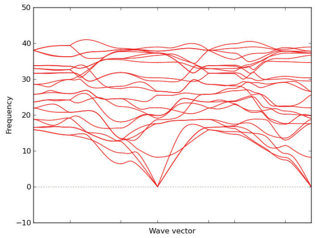

G. *P*4_2/*m*-CN

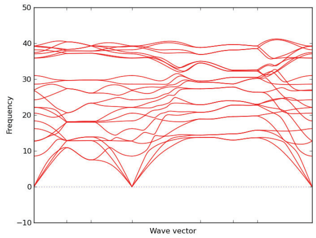

H. *Pnnm*-CN

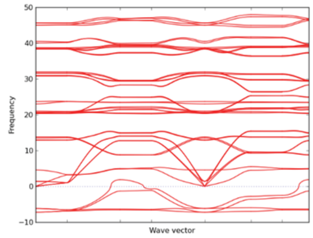

I. graphitic-C_3N_4 (*Pm*2, vdW)

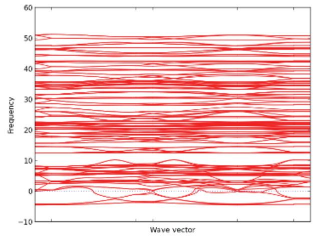

J. graphitic-C_3N_4 ($I4_1md$, vdW)

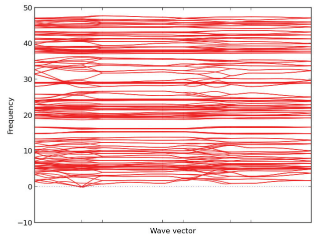

K. s-triazine-C_3N_4

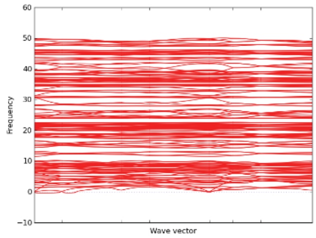

L. s-heptazine-C_3N_4 (*Cc*, vdW)

Supplementary Figure S2 | Phonon dispersion curves of stable structures (A-H), graphitic-C_3N_4 (I-J) and *Cc*-C_3N_4 (K-L) in C-N system at 0 GPa.

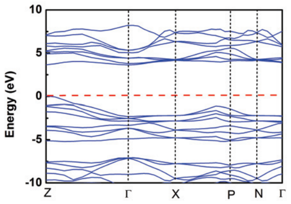

A. $I2d$-CN_2

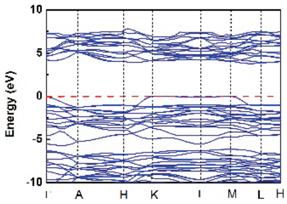

B. $P31c$-C_3N_4

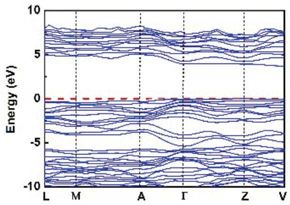

C. Cm-C_3N_4

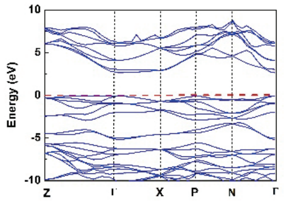

D. $I2m$-C_3N_4

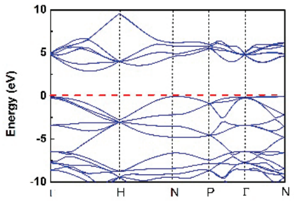

E. $I3d$-C_3N_4

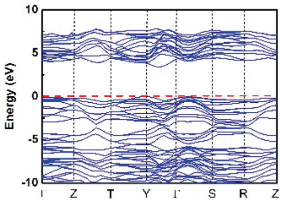

F. $Cmc2_1$-C_3N_4

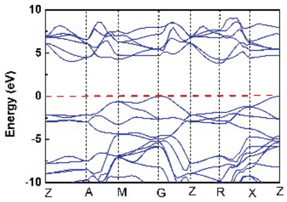

G. $P4_2/m$-CN

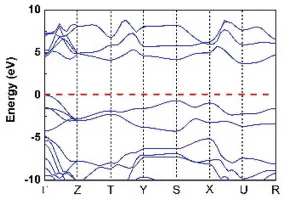

H. $Pnnm$-CN

Supplementary Figure S3 | Band structures of stable structures in C-N system at 0 GPa.

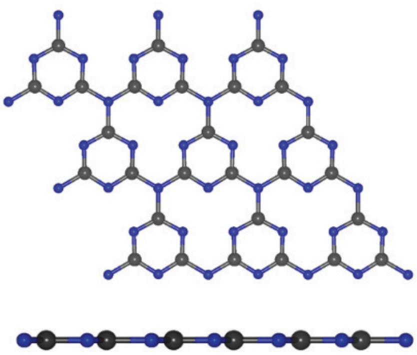

A. Graphitic-C_3N_4

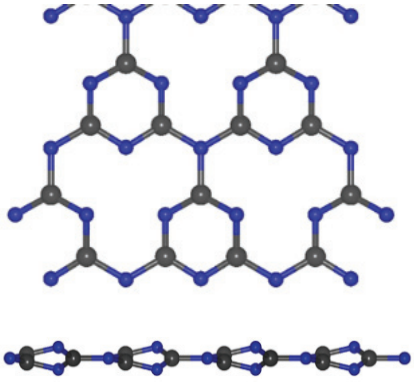

B. *Pbcn*-C_3N_4

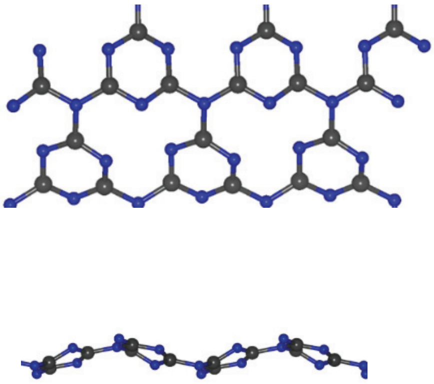

C. *P*-C_3N_4

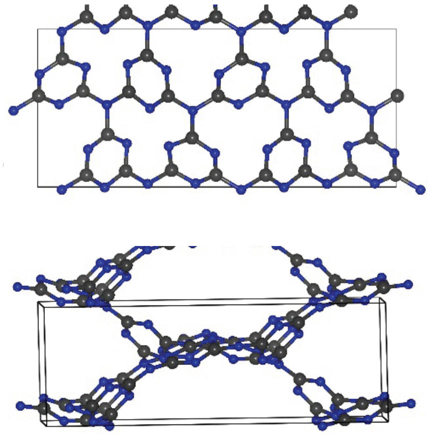

D. *Cc*-s-triazine-C_3N_4

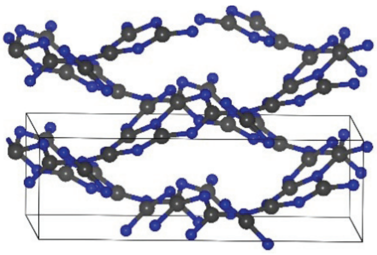

E. *Cc*-s-heptazine-C_3N_4

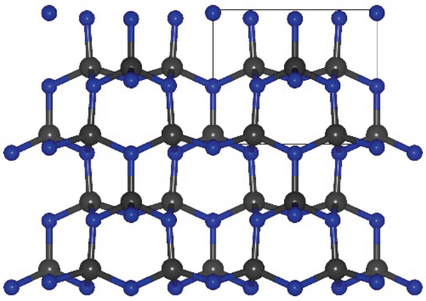

F. *Pmn*2_1-C_3N_4

Supplementary Figure S4 | One layer of (A). Graphitic-C_3N_4, (B). *Pbcn*- C_3N_4, (C). *P*- C_3N_4 looking from different direction. (D) Crystal structure of *Cc*-s-triazine-C_3N_4 and (E) *Cc*-s-heptazine-C_3N_4 and (F) *Pmn*2_1-C_3N_4. Black (big) and blue (small) spheres denote C and N atoms, respectively.

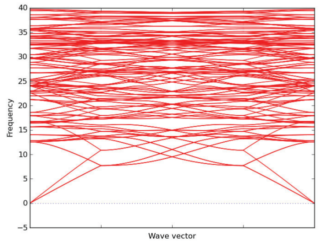

A. $F3m$-$C_{11}N_4$

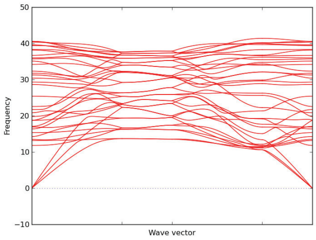

B. $C2/m$-C_2N

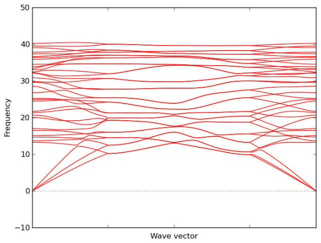

C. $P2_1/c$-C_2N

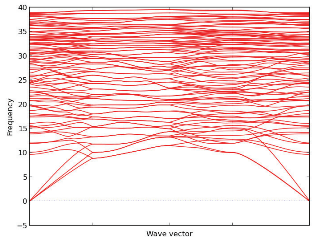

D. Cm-$C_{11}N_4$

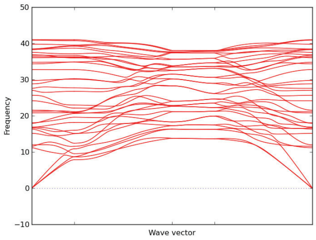

E. $Pmn2_1$-C_2N

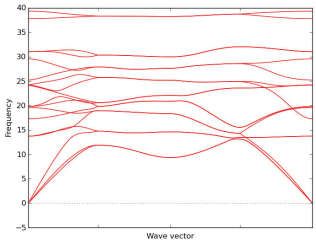

F. $Pn2$-CN_2

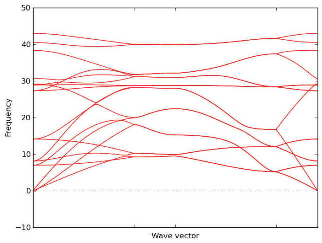

G. $Im2$-CN_2

Supplementary Figure S5 | Phonon dispersion curves of top 5 hardest structures (based on macroscopic model and microscopic model) in C-N system at 0 GPa. (The other three structure's results can be found in Supplementary Figure 2)

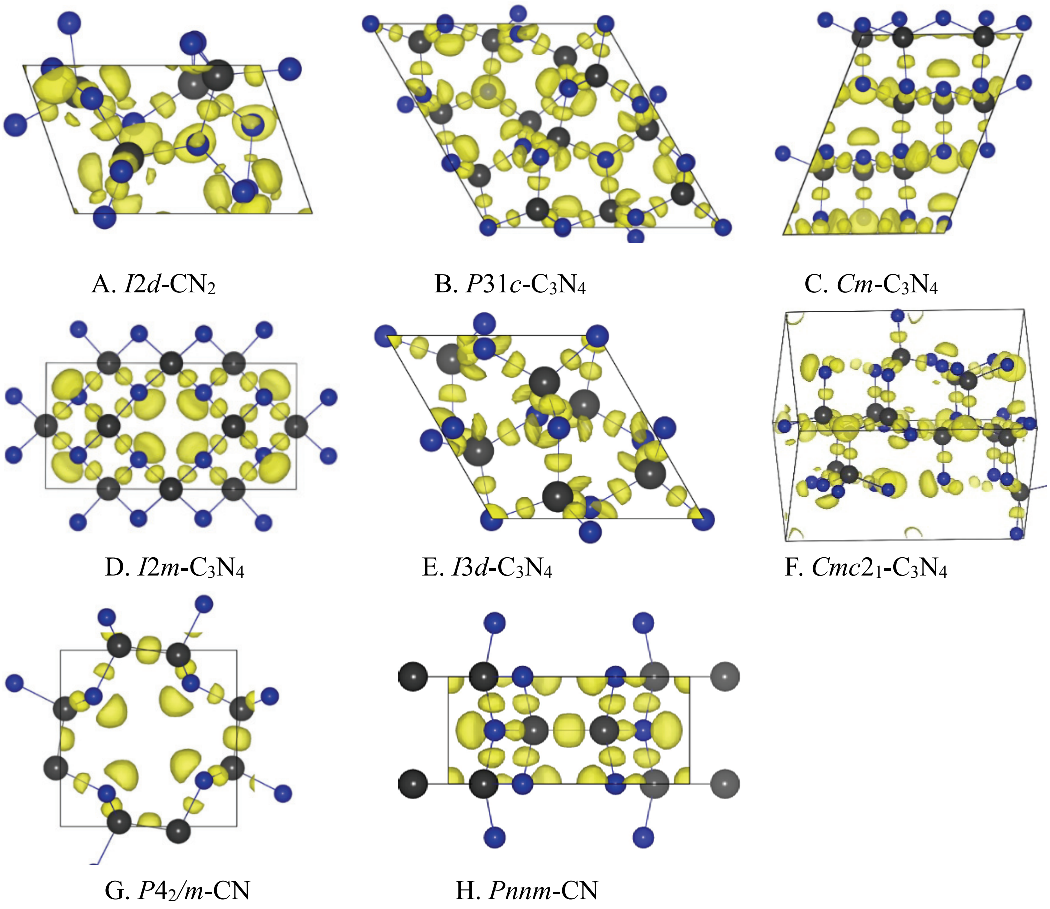

Supplementary Figure S6 | Electron localization function of stable structures in C-N system at ELF= 0.85.

Supplementary Table S1 | Elastic constants of the stable structures at 0 GPa, including diamond's results for comparison.

	c_{11}	c_{22}	c_{33}	c_{44}	c_{55}	c_{66}	c_{12}	c_{13}	c_{14}	c_{15}	c_{16}	c_{23}	c_{25}	c_{35}	c_{46}
Diamond	1054			562			125								
$I2d$-CN_2	741		1187	363		334	206	139							
$P31c$-C_3N_4	834		894	315			186	129	23						
Cm-C_3N_4	938	805	843	311	290	298	79	90		-4		132	28	-17	25
$I2m$-C_3N_4	699		768	416		418	208	192							
$I3d$-C_3N_4	789			472			261								
$Cmc2_1$-C_3N_4	996	883	778	300	285	309	76	45				118			
$P4_2/m$-CN	534		1139	318		225	214	99			32				
$Pnnm$-CN	1187	647	510	373	275	441	141	80				194			

Supplementary Table S2 | Band gap and piezoelectricity of stable structures in the C-N system at 0 GPa.

	Eg		Piezoelectric	d_{14}	d_{15}	d_{22}	d_{31}	d_{33}	d_{36}
	(eV)			10^{-12}(C/N)					
$I2d$-CN_2	3.57	indirect	Yes	-0.17					0.03
$P31c$-C_3N_4	3.78	indirect	Yes		-0.14	-0.33	-0.09	0.20	
Cm-C_3N_4	3.74	indirect	Yes		0.82		0.50	-1.18[a]	
$I2m$-C_3N_4	2.75	direct	Yes	1.88					1.29
$I3d$-C_3N_4	2.91	indirect	Yes	0.25					
$Cmc2_1$-C_3N_4	3.43	indirect	Yes		-0.41		-0.12	-0.42	
$P4_2/m$-CN	3.81	indirect	No						
$Pnnm$-CN	3.71	indirect	No						

[a] This value is comparable with that of α-SiO_2, which is a typical piezoelectric crystal and has d_{11} = 2.31 x 10^{-12} C/N, d_{14} = -0.73 x 10^{-12} C/N [1]

[1] Kittel C. Introduction to Solid State Physics. John Wiley & Sons, Inc. , 1976.

Supplementary Table S3 | Hardness (GPa), computed by microscopic model and macroscopic model, elastic moduli (GPa) and Poisson's ratio *v* for diamond and the top 5 hardest (meta)stable structures. Data are given at zero pressure.

		B	*G*	*E*	*v*	H(Oganov)	H(Chen)
			Top 5 of the Oganov model				
*Pn*2-CN_2	No.118	364	366	822	0.1232	87.8	60.7
*Im*2-CN_2	No.119	395	283	686	0.2105	86.0	33.9
*I*2*d*-CN_2	No.122	398	352	815	0.159	85.6	50.3
*F*3*m*-$C_{11}N_4$	No.216	390	428	940	0.0985	83.8	74.1
*I*3*d*-C_3N_4	No.220	437	374	873	0.1666	83.8	50.5
			Top 5 of the Chen model				
*F*3*m*-$C_{11}N_4$	No.216	390	428	940	0.0985	83.8	74.1
*C*2/*m*-C_2N	No.12	387	424	931	0.0991	67.4	73.5
P2_1/*c* -C_2N	No.14	384	418	921	0.1004	66.9	72.6
Cm-$C_{11}N_4$	No.8	386	417	919	0.1033	82.9	71.5
Pmn2_1-C_2N	No.31	367	397	875	0.1027	56.9	69.6
			Results for diamond				
Diamond	No.227	435	521	1116	0.0001	89.2	92.9

Supplementary Table S4 | Elastic constants of diamond and top 5 hardest (meta)stable carbon nitrides. Data are given at zero pressure.

	c_{11}	c_{22}	c_{33}	c_{44}	c_{55}	c_{66}	c_{12}	c_{13}	c_{15}	c_{23}	c_{25}	c_{35}	c_{46}
				Top 5 of the Oganov model									
*Pn*2-CN_2	1018		456	390		505	67	200					
*Im*2-CN_2	931		**1568**	214		125	12	55					
*I*2*d*-CN_2	741		1187	363		334	206	139					
*F*3*m*-$C_{11}N_4$	883			472			144						
*I*3*d*-C_3N_4	789			472			261						
				Top 5 of the Chen model									
*F*3*m*-$C_{11}N_4$	883			472			144						
*C*2/*m*-C_2N	788	1210	822	450	521	443	124	189	149	109	46	160	123
P2_1/*c* -C_2N	1288	1188	695	326	324	543	135	15	86	74	4	0	16
Cm-$C_{11}N_4$	1002	991	985	414	383	398	74	76	-19	99	-47	58	-58
Pmn2_1-C_2N	811	733	1200	404	410	380	92	106		118			
				Results for diamond									
Diamond	1054			562			125						

Supplementary Table S5 | Comparison of free energy between new predictions and the prior proposals.

Structures	Space group	Free energy* per formula (eV)
New_ s-heptazine-unit-3D-C_3N_4	*Cc*	-60.0204
New_s-triazine-unit-3D-C_3N_4	*Cc*	-59.8212
Best six structures described in the review of Kroke [2]		
3D-ths-C_3N_4		-59.6681
g-$[(C_6N_7)(N)]n$		-59.6038
3D-srs-C_3N_4		-59.1845
g-C_3N_4	*Pm*2	-59.2387
α-C_3N_4	*P*31*c*	-58.6794
β-C_3N_4	$P6_3/m$	-58.4137
Best structure predicted by Kroll in 2009 [3]		
h-$ThSi_2$.Heptazine	*P*1	-59.9812

* The free energy have considered Van der waals corrections.

[2] Kroke, E. & Schwarz, M. Novel group 14 nitrides. *Coord. Chem. Rev.* **248**, 493-532 (2004).

[3] Gracia, J. & Kroll, P. *J. Mater. Chem.* **19**, 3013-3019 (2009).

Supplementary data S1 | Stable structures of C-N system at high pressure:

***I2d*-CN_2** structure has 4 f.u. in the unit cell. This structure at 60 GPa has parameters $a = b = 6.190$ Å, $c = 3.335$ Å, and $\alpha = \beta = \gamma = 90°$, with two nonequivalent atoms C and N occupying the Wyckoff 4j (0.7500, 0.4422, 0.3750) and 4j (0.0666, 0.8126, 0.7847) sites, respectively.

***P*31*c*-C_3N_4** structure at 30 GPa has lattice parameters $a = 6.361$ Å, $c = 4.637$ Å, $\alpha = \beta = 90°$, and $\gamma = 120°$, with C atoms occupying the Wyckoff 1c (0.4358, 0.5162, 0.1985) and 1c (0.9113, 0.1664, 0.9910) positions, and N at 1c (0.3974, 0.3461, 0.9688), 1c (0.9907, 0.3134, 0.2452), 1a (0.0, 0.0, 0.9907) and 1b (0.6667, 0.3333, 0.6162) sites.

***Cm*-C_3N_4** structure is stable at 68.3-98.4 GPa. This structure at 70 GPa has parameters $a = 11.885$ Å, $b = 4.685$ Å, $c = 4.057$ Å, $\alpha = \gamma = 90°$, and $\beta = 103.426°$, with C atoms occupying the Wyckoff 4b (0.0021, 0.7511, 0.9569), 2a (0.9995, 0.0, 0.4607), 2a (0.1687, 0.5000, 0.2343), 4b (0.1678, 0.2508, 0.7356), 2a (0.8340, 0.5000, 0.0092), and 4b (0.8333, 0.7500 0.5128) positions, and N at 4b (0.1264, 0.2624, 0.0339), 2a (0.1208, 0.0, 0.5636), 2a (0.1299, 0.5000, 0.5349), 4b (0.7900, 0.2601, 0.8128), 2a (0.7911, 0.0, 0.3261),2a (0.7972, 0.5000, 0.3133), 2a (0.9567, 0.5000, 0.0691),2a (0.9652, 0.0, 0.1077), and 4b (0.9568, 0.7622, 0.6022) sites.

***I2m*-C_3N_4** structure at 100 GPa has lattice parameters $a = 3.262$ Å, $c = 6.524$ Å, and $\alpha = \beta = \gamma = 90°$, with C atoms occupying the Wyckoff 2b (0.5000, 0.5000, 0.0) and 2d (0.5000, 0.0, 0.7500) positions, and N at 8i (0.2579, 0.2579, 0.1262) sites.

***I3d*-C_3N_4** structure at 190 GPa has lattice parameters $a = 4.989$ Å, with C atoms occupying the Wyckoff 4b (0.5000, 0.2500, 0.1250) positions, and N at 4c (0.7236, 0.2236, 0.2764) sites.

***Cmc*2₁-C₃N₄** structure at 0 GPa has lattice parameters $a = b = 4.715$ Å, $c = 8.529$ Å, and $\alpha = \beta = 90°$, $\gamma = 117.544°$, with C atoms occupying the Wyckoff 2b (0.6984, 0.2059, 0.0960), 2a (0.2028, 0.2028, 0.0162), 2a (0.5485, 0.5485, 0.8426) and 2b (0.5466, 0.0513, 0.7613) positions, and N at 2a (0.0215, 0.0215, 0.0308), 2a (0.4978, 0.4978, 0.5063), 2a (0.2647, 0.2647, 0.3358), 2a (0.7567, 0.7567, 0.3439), 2b (0.5145, 0.0249, 0.0981) and 2b (0.7180, 0.2464, 0.7564) sites.

***P*4_2*/m*-CN** structure at 15 GPa has lattice parameters $a = 4.583$ Å, $c = 2.352$ Å, and $\alpha = \beta = \gamma = 90°$, with C atoms occupying the Wyckoff 4j (0.0265, 0.6672, 0.5000) positions, and N at 4j (0.2629, 0.1893, 0.5000) sites.

***Pnnm*-CN** structure at 30 GPa has lattice parameters $a = 2.333$ Å, $b = 3.860$ Å, and $c = 5.129$ Å, with C atoms occupying the Wyckoff 1g (0.5000, 0.4464 0.3545) positions, and N at 1g (0.5000, 0.2517, 0.8135) sites.

Supplementary data S2 | Top five of the hardest structures in C-N system (zero pressure):

***F*3*m*-$C_{11}N_4$** structure has lattice parameters $a = 7.065$ Å, with C atoms occupying the Wyckoff 24g (0.7500, 0.2500, 0.4927), 12e (0.1240, 0.8760, 0.6240), and 6b (0.5000, 0.0, 0.0) positions, and N at 12e (0.1296, 0.8704, 0.1296) sites.

***C2/m*-C_2N** structure has lattice parameters $a = 6.691$ Å, $b = 2.418$ Å, $c = 4.510$ Å, $\alpha = \gamma = 90°$, and $\beta = 104.589°$, with C atoms occupying the Wyckoff 4i (0.2350, 0.0, 0.8944), and 4i (0.9973, 0.0, 0.6731) positions, and N at 4i (0.8900, 0.5000, 0.7230) sites.

***P*2_1*/c*-C_2N** structure has lattice parameters $a = 3.548$ Å, $b = 4.178$ Å, $c = 5.051$ Å, $\alpha = \gamma = 90°$, and $\beta = 109.227°$, with C atoms occupying the Wyckoff 1e (0.2922, 0.6620 0.7370), and 1e (0.9596, 0.1690, 0.5389) positions, and N at 1e (0.3358, 0.3306, 0.6617) sites.

***Cm*-$C_{11}N_4$** structure has parameters $a = 8.663$ Å, $b = 5.000$ Å, $c = 4.336$ Å, $\alpha = \gamma = 90°$, and $\beta = 109.565°$, with C atoms occupying the Wyckoff 4b (0.1193, 0.2594, 0.1604), 2a (0.8616, 0.0, 0.1606), 2a (0.1161, 0.5000, 0.6452), 2a (0.8025, 0.0, 0.7861), 4b (0.0542, 0.7461, 0.7766), 2a (0.1188, 0.0, 0.6530), 4b (0.8625, 0.2534, 0.6440), and 2a (0.0525 0.0, 0.2808) positions, and N at 4b (0.8011, 0.7619, 0.2821), 2a (0.0611, 0.5000, 0.2836), and 2a (0.7992, 0.5000, 0.7410) sites.

***Pmn*2_1-C_2N** structure has parameters $a = 5.409$ Å, $b = 5.565$ Å, $c = 2.408$ Å, and $\alpha = \beta = \gamma = 90°$, with C atoms occupying the Wyckoff 2a (0.9540, 0.7989, 0.0), 2a (0.4407, 0.6090, 0.0), 2a (0.1575, 0.1530, 0.5000), and 2a (0.0399, 0.6549, 0.5000) positions, and N at 2a (0.1009, 0.0202, 0.0), and 2a (0.3043, 0.6545, 0.5000) sites.

***Pn*2-CN_2** structure has parameters $a = b = 3.337$ Å, $c = 3.195$ Å, with C atoms occupying the Wyckoff 2a (0.5000, 0.5000, 0.5000) positions, and N at 4g (0.6431, 0.8569, 0.7500) sites.

***Im*2-CN**$_2$ structure has parameters $a = b = 2.468$ Å, $c = 5.990$ Å, with C atoms occupying the Wyckoff 2d (0.5000, 0.0000, 0.7500) positions, and N at 4e (0.5000, 0.5000, 0.8881) sites.

Supplementary data S3 | Other interesting structures mentioned in the paper

***Pbcn*-C$_3$N$_4$** structure at 70 GPa has parameters $a = 7.882$ Å, $b = 4.506$ Å, $c = 4.691$ Å, and $\alpha = \beta = \gamma =$ 90°, with C atoms occupying the Wyckoff 4c (0.2588, 0.0, 0.2500), and 8d (0.4802, 0.2607, 0.7331) positions, and N at 8d (0.8177, 0.7517, 0.8131), 4c (0.5694, 0.5000, 0.7500), and 4c (0.0673, 0.5000, 0.7500) sites.

***P*-C$_3$N$_4$** structure at 0 GPa has parameters $a = 4.669$ Å, $b = 7.900$ Å, $c = 8.900$ Å, $\alpha = 78.182°$, $\beta =$ 96.662°, $\gamma = 90.665°$, with C atoms occupying the Wyckoff 2i (0.6485, 0.8364, 0.2890), 2i (0.9093, 0.5740, 0.3030), 2i (0.9030, 0.0468, 0.2055), 2i (0.1572, 0.3268, 0.2488), 2i (0.4246, 0.0584, 0.2348), and 2i (0.3751, 0.5761, 0.3330) positions, and N at 2i (0.9278, 0.4118, 0.2213), 2i (0.9026, 0.8872, 0.2319), 2i (0.6676, 0.1187, 0.1713), 2i (0.1619, 0.1422, 0.2170),2i (0.6389, 0.6658, 0.3203), 2i (0.6389, 0.6658, 0.3203), 2i (0.1243, 0.6634, 0.3672), and 2i (0.3862, 0.4043, 0.3065) sites.

***Pna*2$_1$-C$_3$N$_4$** structure at 0 GPa has parameters $a = 9.091$ Å, $b = 5.368$ Å, $c = 6.815$ Å, $\alpha = \beta = \gamma = 90°$, with C atoms occupying the Wyckoff 1a (0.6414, 0.0037, 0.6487),1a (0.4907, 0.1667, 0.4224), and 1a (0.7270, 0.2616, 0.4148) positions, and N at 1a (0.5044, 0.0180, 0.5750),1a (0.5927, 0.3124, 0.3437),1a (0.7588, 0.1093, 0.5669), and 1a (0.3505, 0.1408, 0.3214) sites.

***Cc*-C$_3$N$_4$ (s-triazine)** structure at 0 GPa has parameters $a = 6.265$ Å, $b = 8.014$ Å, $c = 9.223$ Å, $\alpha =$ 95.948°, $\beta = 70.144°$, and $\gamma = 107.764°$, with C atoms occupying the Wyckoff 1a (0.4155, 0.1024, 0.4915),1a (0.7248, 0.3333, 0.3908),1a (0.6991, 0.0759, 0.2720), 1a (0.8785, 0.0769, 0.9945), 1a (0.9299, 0.3108, 0.8617), and 1a (0.1083, 0.1074, 0.7474) positions, and N at 1a (0.5148, 0.2760, 0.4980),1a (0.4923, 0.9980, 0.3739),1a (0.8370, 0.2387, 0.2815), 1a (0.7811, 0.9829, 0.1345), 1a (0.8354, 0.5206, 0.3859), 1a (0.8025, 0.2138, 0.9889),1a (0.0409, 0.0234, 0.8801), and 1a (0.0711, 0.2587, 0.7316) sites.

***Cc*-C$_3$N$_4$ (s-heptazine)** structure at 0 GPa has parameters $a = 4.170$ Å, $b = 6.939$ Å, $c = 11.388$ Å, $\alpha =$ 92.365°, $\beta = 97.892°$, and $\gamma = 72.512°$, with C atoms occupying the Wyckoff 1a (0.5958, 0.9151, 0.2395),1a (0.6963, 0.5449, 0.2500),1a (0.4289, 0.2879, 0.5651), 1a (0.0304, 0.5834, 0.9187), 1a (0.5428, 0.8853, 0.8937), and 1a (0.6680, 0.22490, 0.2363) positions, and N at 1a (0.4182, 0.9497, 0.3313),1a (0.6989, 0.5601, 0.3683),1a (0.5634, 0.2540, 0.3493), 1a (0.3171, 0.1512, 0.4993), 1a (0.5778, 0.4183, 0.5341), 1a (0.3919, 0.2806, 0.6883),1a (0.7141, 0.0433, 0.1871), and 1a (0.7191, 0.3786, 0.1840) sites.(with vdW)

***Pmn*2$_1$- C$_3$N$_4$** structure at 0 GPa has parameters $a = 4.055$ Å, $b = 4.274$ Å, $c = 4.942$ Å, and $\alpha = \beta = \gamma =$ 90°, with C atoms occupying the Wyckoff 4b (0.4294, 0.3374, 0.2501), and 2a (0.9312, 0.1765, 0.0) positions, and N at 2a (0.0694, 0.7919, 0.5000), 2a (0.0252, 0.8482, 0.0), and 4b (0.0644, 0.3469, 0.7701) sites.
